# The Connexion between Physiology, Psychology, Natural Theology, and Other Sciences

**Published:** 1849-04-01

**Authors:** George Ogilire

**Affiliations:** Lecturer on the Institutes of Medicine in the University and King's College, Aberdeen


### ON THE CONNEXION BETWEEN PHYSIOLOGY, PSYCHOLOGY, NATURAL THEOLOGY, AND OTHER SCIENCES.

BY GEORGE OGILIRE, M.D.

*Lecturer on the Institutes of Medicine in the University and King's College, Aberdeen.**

It is proposed, in the present discourse, to point out the relation in which physiology, considered as a philosophical pursuit, stands to some other branches of science; and, in particular, it is intended to show that it stands related, in one respect, to the physical, and in another, to the metaphysical sciences, occupying, as it were, a station intermediate between them.

Physical science is concerned with matter, as influenced by various forces—such as gravitation, chemical affinity, heat, electricity, &c.—whose tendency is to bring it into a state of motion, sensible or malicular. When, however, we speak of matter as influenced by certain forces, we must be understood as merely using a conventional form of expression; for we have no reason to suppose the influencing force to be distinct from the induced state of change; thus, sound, which may be spoken of as a force, is nothing else than a peculiar vibratory condition of the molecules of the sounding medium, and the same remark probably applies to heat, light, electricity, &c. Nor can we attach a different meaning to the expression when we speak of the body, or any part of it, as being under the influence of the vital force. All that is implied is, that the body is in a peculiar state of change, differing from matter not vitalized, much as a sounding body does from one that is mute. We may consider, therefore, vitality as a force co-ordinate with any of the above-mentioned physical forces, as, for instance, heat, or electricity—in other words, that a vitalized differs from an inanimate body, much as a heated does from a cold one, or an electrified from a non-electrified one. And among these forces, the relation of vitality is closer to electricity than to any other one, though it is now generally admitted that they are only analogous, not identical. Just as a piece of iron placed in a certain relation to the electrical current, without altering either its physical or its chemical constitution, becomes a magnet, and acquires new properties different from those

* Read before the Medico-Chirurgical Society, Aberdeen, March 1st, 1849.

of other pieces not so situated, so certain organic compounds, when placed within the influence of the living tissues, become assimilated to them, and endued with life; acquiring new properties, and a capability of assuming new forms very different from what they previously possessed, having undergone, at the same time, it may be, scarce any appreciable alteration, either in chemical or physical characters. Thus the plasma liquor sanguinis appears nearly undistinguishable in either of these respects from some forms of fluid albumen, from which (as they are capable of being used for food) it may have been formed. Yet it has acquired new properties; for while the albumen tends only to resolve itself by putrefaction into its component elements, the fluid fibrine of the blood, immediately on being effused from the vessels, becomes solid, often assuming a distinctly fibrous structure; and the coagulum or solid so formed, if remaining in relation with the living tissues, will develop nuclei and cells, to be afterwards converted into a substance identical in properties and appearance with some of the natural and original tissues of the body. But not only is there thus a general analogy of action between the vital and electrical forces, but the action of the one may even excite that of the other. Thus electricity will not only excite contraction of the muscular fibre if applied directly to its substance as many other irritants will do, but also, if made to act upon the motor nerve supplying it, or even upon the nervous centre from which the nerve is derived; and the natural actions of many parts of the body may be excited by means of electricity, when the influence on them of the nervous system has been in various ways obstructed. Then, again, we find that it is by means of the nervous system that the action of the electrical organs found in certain fishes is subjected to the will of the animal. The act of volition causes a certain change in the nervous centre appropriated to this function, and the resulting influence, or nervous force, being transmitted along the nerve which supplies the apparatus, causes there a manifestation of electrical action.

The student of physical philosophy will not fail here to remark, that the researches of Faraday, and others, have shown that a similar reciprocity of action prevails among the purely physical forces. Thus heat induces electrical disturbance in the tourmaline, and in certain metallic combinations, and light induces magnetism, while the electro-magnetic current will, under certain circumstances cause the deflection of a ray of light.

But we ground the relationship of physiology and physical philosophy, not only on the vitalized condition of the organism being a state closely analogous to those conditions of inanimate matter which are treated of by the latter science, but also on the essential identity, both in chemical and in mechanical properties, between the living tissues and inanimate matter. So much is this the case, that many of the processes going on within the living body are entirely explicable by the laws known to regulate such changes in inorganic matter—*e. g.*, the association of movements, the propulsion of the blood, and of the contents of the alimentary canal, &c., some parts of the processes of digestion and arterialization; though, at the same time, as already intimated, there are others to be referred apparently to a force (that of vitality) different from, though bearing an analogy to, those concerned in the production of simply physical phenomena, (*e. g.*, the influence of the nervous system in causing contraction of the muscular fibre, as well as assimilation, nutrition, secretion, reproduction, &c.)

And there are other functions, again, of a complex nature, intermediate, as it were, between the two classes just noticed; it must, however, be admitted that the tendency of modern investigation is to extend the operation of physical at the expense of the purely vital phenomena.

I proceed next to speak of the relation in which physiology and pathology stand to the metaphysical group of sciences.

Under this group I comprehend psychology, or the philosophy of the human mind, and theology, or that of the Divine Mind; as also ethics, or that science which treats of the relations in which we stand to that Divine Mind and to each other. Now, as physiology and pathology are related to physics and chemistry, inasmuch as the living body about which they concern themselves is merely a form of matter subject in general to the same physical and chemical laws as matter not vitalized, as they are related to the philosophy of electricity and the cognate sciences; inasmuch as the matter of which the living body is composed has its constitution so influenced by its vitality as that it has properties differing from those of other and inanimate matter, much in the same way as a magnet has properties differing from those of other pieces of iron not magnetized; so in the case of our own species are they related to those of the present group, inasmuch as man's material body is influenced, not only by the force of vitality, but also by his immaterial mind.

I proceed, of course, upon the understanding that the nature of man is admitted to be twofold—that his body is the residence and instrument of an immaterial soul, which acts upon, and is acted upon, by the former through the intervention of the nervous system.

Such is the view, indeed, taken by almost all who have written on the subject of human nature. Not that I mean to say there is perfect unanimity on the subject, for there is a certain class of minds that delight in holding opinions opposed to those of the rest of mankind, solely, as it would seem in many cases, from the love of singularity. Thus we find some of the disciples of Bishop Berkeley denying the existence of matter, and calling in question the evidences of our senses, because they do on some rare occasions deceive us, and because we cannot have any demonstrative proof of the credibility of their testimony. And, in like manner, some authors deny the existence of mind as distinct from matter, holding that the mental phenomena exhibited by the higher animals are merely the necessary result of certain molecular changes going on in their brains—in other words, not that the brain is the instrument of the mind, but that mind is a function of the brain, just as contractility is a function of muscle.

A little consideration, however, will show that our belief in the existence of mind and of matter rest essentially on the same foundation. We believe in the existence of our own bodies, (independently of the evidence of our external senses,) from an intuitive conviction in the truth of the impressions derived from our internal sensations. We believe in the existence of bodies, external to us, from an intuitive conviction of the trustworthiness of the evidence of our senses; and, in like manner, we believe in the existence of our own minds, as distinct from our bodies, from an intuitive conviction that we possess the power of thinking, willing, feeling, and remembering, and from a further conviction that these are powers altogether different in kind from those residing in any form of matter, which convictions we denominate consciousness; and we believe in the being of other spiritual existences, as much external to our own minds as the objects of sense are external to our own bodies, (and, in particular, of one Supreme Being,) originally, I conceive, from an undefined but irresistible conviction of their existence spontaneously arising in the mind. That such an intuitive conviction does exist in our minds is shown in various ways. "Most people are conscious of it in themselves, especially in the silence and darkness of night, or in other similar situations, when from the withdrawal of the more palpable impressions made on the mind through the external senses, it is more free to be affected by its internal convictions."* This is particularly seen in the case of children. Why is it that a child

* Williams' Study of Gospels.

cries, and is afraid when it finds itself alone in the dark? Why, but from a strong though undefined conviction of the presence of unseen beings. Now, the constitution of a child's mind does not differ essentially from that of an adult. All the same powers are there, though some of them are in a rudimentary state; and as we study the development of the corporeal organs in the embryonic state, in order to arrive at an understanding of their real nature in the full-grown body, so I am persuaded will the study of the comparatively simple mental phenomena of children be found, when diligently pursued, (which it has never been as yet,) to throw great light on the more complex processes in the minds of adults; and I hold it perfectly legitimate to argue from the one to the other in the case now before us. This view is further borne out by the common belief of mankind in the existence of spiritual beings, for there is not, nor ever has been, any race of men, however ignorant and barbarous, that has not had a religious or mythological system in reference to such beings. Such an intuitive belief it is which has led nations, who have in any degree cherished the spirit of poetry, to people their woods, waters, their hills and valleys, their fountains and rivers, with nymphs or elves, or other supernatural beings; and it is a perversion of this natural tendency in the human mind which has led to the polytheism and superstition of heathen countries. It has been left for the refinement of modern philosophy to deny, by the doctrine of materialism, that the corporeal body, the *microcosm*, is animated and controlled by a living soul, and by the system of pantheism, (the only form of atheism which can be sincerely embraced by a rational being,) that the universe, the *macrocosm*, is the work, the subject, and the property of the Omnipotent and self-existent mind, on whose continued volition depends its continued existence; to maintain, in other words, that the incommunicable names by which we distinguish the Great First Cause and Father of all, are mere conventional terms to express the life of the world, the peculiar order or system of laws which we observe to prevail throughout the universe.

I maintain, therefore, that our belief in the existence of anything whatsoever—of our bodies as well as our minds—of ourselves or of things external to ourselves, whether they be corporeal or spiritual, is grounded on certain intuitive convictions which we cannot, indeed, explain, but whose existence we must admit as an ultimate fact, which are as much part of our whole nature as the contractibility of muscle on the reflex and other functions of the nervous system are of the corporeal part of it.

There is, indeed, much truth in the remark made by some writers, that the evidence for the existence of mind is even stronger than that for the existence of matter, though it may be less palpable. There are, however, other arguments to the same effect, as, for instance, the inadequacy, in many cases, of the morbid changes in the brain, to account for the phenomena of insanity. Admitting, therefore, that the popular view of the constitution of man is also the philosophical one, it follows that there must be a close relationship between the sciences of physiology and psychology, inasmuch as they treat of the two elements, corporeal and mental, entering into the constitution of our nature, especially when we approach that department of physiology which treats of the structure of the brain, the instrument by which the mind acts on the body, and to consider its pathology. So great, indeed, is the reciprocal influence of mind and body, that it is impossible to treat satisfactorily of the one without constant reference to the other. I am no disciple of Gall and Spurzheim; I consider their system faulty and defective, yet (while I have no sympathy for the materialistic opinions in the minds of many associated with the name) I unhesitatingly pronounce myself a phrenologist in the true sense of the word; and I hold that any system of psychology which does not take into view the structure, functions, development, and comparative anatomy of the brain, is as defective as a system of physiology which omits all mention of the influence of imagination, hope, fear, surprise, &c., on the bodily organs.

Let us pass now from the consideration of the constitution of the mind of man to that of the laws regulating his moral relations, which also have no unimportant bearing on physiology. This will appear from considering on what these relations are founded. They are founded, as it seems to me, on certain intuitive convictions,—as, for instance, of the existence of a Supreme Being, and in some degree also of his attributes, of right and wrong, of future retribution, and perhaps also of the immortality of the soul,—and on certain innate affections, such as devotion, love of virtue, of truth, of justice, &c., compassion, and the like. It is mainly in these, its moral attributes, that the mind of man differs from that of the inferior animals; for that the more highly organized among these do possess minds, we can hardly deny, seeing they display unequivocal indications both of intellect and feeling, but they are totally deficient in the moral intuitions and feelings above mentioned. Intuitions, and feelings, and propensities they have, differing in different cases, according to the habits of the species, and often of a very remarkable kind, being what we denominate instinct, but they are of a lower nature, being mostly in reference to bodily wants and sensations, and even when they rise somewhat above this, as in the dog and elephant, they are still totally different in kind from those proper to the human mind. Thus we have no reason to believe that the dog can raise in his mind the idea of any being higher than his master, nor has he any conscience, properly so called; his master's variable will being to him the sole rule of right and wrong. Hence the expression of the poet Burns, that "man is the god of the dog."

Now, among the evidences of the close connexion between the moral and material parts of our nature, may be included the fact, that, in a large proportion of cases at least, whatever is detrimental to the former is so also to the latter. Our moral feelings, for instance, prescribe to us certain laws of chastity, temperance, sobriety, &c., which cannot be infringed without at the same time deteriorating our moral state, and inducing greater or less disorder in the bodily functions. And, on the other hand, sickness, lassitude, pain, and other pathological states of the body, tend very materially to interfere with the due exercise of our moral powers, as is evidenced by the petulance, indolence, and selfishness, almost all invalids are prone to manifest. "It is very evident," says Mr. Williams, "that there are states of bodily ailment or disorder, to be removed by medical treatment, and arising from obvious physical causes, which are closely connected with spiritual sins. Such are thoughts of gloominess, discontent, unkindness, which mere bodily disarrangement gives rise to; so that it is impossible to say how intimate the connexion may be between bodily temperament and spiritual influences."*

On this intimate relation and reciprocal influence of the moral and material parts of our nature, is founded a great part of the penitential discipline of the Christian religion; nor can any system of education be thoroughly efficient in which they are lost sight of.

The universal and almost involuntary habit of judging of a man's moral character by the cast and expression of his features, is another indication of the same truth; and this holds, in a degree, of races as well as of individuals, for there is such a thing as a national physiognomy, which generally indicates correctly enough some corresponding trait in the national character. How true an indication is the degraded physiognomy of the negro race of the state of ignorance, barbarism, and vices of various kinds, in which they have re-

* Study of Gospels, p. 302.

mained for ages; or, on the other hand, the elevated cast of features in the European, of the exalting influence of Christianity and civilization.

A curious parallel may be drawn between the bodily and moral functions, in the tendency of certain morbid states to become hereditary. In the case of bodily disease this is well known to hold of scrofula, gout, &c.; and in like manner (not to speak of the moral deterioration of the whole human family, or of particular races) we see, in particular instances, that it is true also of drunkenness and other vicious habits. It is not, however, the disease, properly speaking, that becomes hereditary, but a tendency to the disease, or a morbid diathesis, as it is termed by pathological writers; a state in which a less exciting cause than would otherwise be required is sufficient to excite an active manifestation of morbid action, but which, nevertheless, by care and exertion on the part of the individual, and by the use of such remedial measures as are put within his reach, the danger may generally, if not always, be warded off. In speaking of drunkenness as becoming in certain cases hereditary, I refer to what is described by some writers as a peculiar form of insanity, under the name of binomania, in which the patient, though brought up, it may be, out of the way of temptation, manifests an extraordinary desire to indulge to excess in alcoholic liquors, whenever they come within his reach, just as one would do who had previously, by numerous acts of indulgence, acquired for himself this propensity. And this leads me now to speak of some other forms of insanity in which morbid affections of the brain are associated with a perversion of the moral faculties. This is a subject of great practical importance, inasmuch as it is often in such cases a matter of the greatest difficulty to determine in how far the patient is to be considered as a responsible agent.

Whether the abstract conviction of right and wrong is ever lost by the human mind, except in cases of extreme idiocy, in which all the other mental faculties are almost entirely absent, I am inclined to doubt, but certainly nothing is more common than for the intellectual powers to be so deranged as to render the patient incapable of determining, in particular cases, as to which of different lines of conduct is right, and which wrong. Or, again, the emotions and passions may at times acquire such force as to constrain the will and, in a manner, compel the person to follow a course which he knows to be wrong. Now both these forms of insanity, in which there is a greater or less perversion of the healthy state of the moral faculties, may arise from a pathological state of the central masses of the nervous system, but they may also arise from causes purely moral: thus we know that, by a long continued succession of wilful violations of the moral law, the intellectual powers are so far deranged, that the person becomes an incompetent judge of the moral value of actions—his conscience becomes seared—he is given over to a strong delusion that he should believe a lie; and at the same time his evil passions acquire such force, that, even should he awake to the mischievous tendency of his course of life, he has no longer the power to resist them. Thus we find that closely analogous states of moral perversion, both as regards the intellect and the passions, may be the result both of pathological states of the body, and of a particular course of moral conduct, which surely implies a very close relation, in certain particulars, between the branches of science occupied with the examination of the functions of the material body, and the moral powers of the mind. The only difference, in fact, between the states of moral insanity (using the term in its widest sense) and confirmed viciousness is, that in the former the intellect and passions are first deranged, and the will is, in consequence, either deceived (as it were) or overled; while, in the latter, the will first goes astray, and in the course of time deranges the due play of the passions and intellectual powers, which then re-act on it in a morbid manner; in other words, insanity is an involuntary viciousness—a voluntary state. This, however, is to be taken with some limitation; for, on the one hand, individuals may, by the force of bad education, and other such causes, be brought into a state of vice almost without any proper consent of their own; while insanity, in the majority of cases, is induced more or less by voluntary transgressions of the moral law. Hysteria is, perhaps, of all such cases, in which a pathological state of the body is associated with moral perversion, the most remarkable and difficult of explanation.

In the phenomena of the dreaming and half-waking state, we have a sort of epitome of those connected, both with the voluntary and involuntary states of moral perversion above alluded to. At times we lose our due discrimination of the propriety of actions—at other times we seem to be constrained to perform certain actions, which all the time we know to be wrong. Who is there that has not at times, when under the impression that he had knowingly, but, as it seemed to him, involuntarily, committed some fearful crime, awaked, and felt thankful when he found that it was only a dream? Now dreams are, in a certain degree, remotely voluntary, in so far, as that a man's habitual character, and even his later thoughts, will give a colour to his dreams; yet, at the same time, dreams are undoubtedly excited and directed mainly by the physiological state of the body at the time, or by the influence on it of external agencies. One other circumstance may be noticed in reference to this subject; that, namely, of which we are assured by revelation—that there is a spiritual body as well as a natural body; but the spiritual is not a different body from the natural, but the same body in a more advanced state of development, bearing to the latter the same relation as the seed to the perfect plant—the egg to the full-grown animal, —the cytoblast to the fully-formed organ developed from it.

On the connexion between physiology and theology I shall not enlarge, as it would lead me into discussions hardly suitable on the present occasion: I will only observe that, though the Divine mind has properly no passions, and cannot be supposed to be conformed to the type of any created intelligence, yet all the ideas we can form of it must be through the medium of, and therefore in some degree assimilated to, the constitution of our own minds. In fact, in Holy Scripture the Divine Being is spoken of in language which, if literally understood, would imply the possession of a body and mind similar to our own.

Perhaps, however, I should not dismiss the subject without some allusion to what is called natural theology, or the evidence of the Divine Being and attributes afforded by the natural sciences—and perhaps by those of anatomy and physiology more than by any others; but I confess I cannot take the same high view of the value of this kind of argument as many modern writers do, as it seems to me defective, both in force and extent. In force, because the fundamental position upon which it is based is one which by no means carries conviction to all minds. This position, of course, is, that every piece of mechanism implies a rational constructor, endowed with power, and wisdom, and goodness, corresponding to the perfection of the work and the good it accomplishes. But the pantheist meets this argument by the assumption, that the machine and the mechanist are one and the same; that the visible universe is in itself divine and self-existent; and that what we bring forward as evidences of design, are merely the necessary results of the mutual re-actions of different parts of this self-existent system. That the real utility of any particular result is not always a demonstrative evidence of design may easily be shown. Thus, what system would be more convenient, or has actually served greater ends of utility, than that of logarithms. How singular it appears, and how like the working of design, that there should be certain numbers bearing such a relation to other numbers, that the simple process of adding together the first can be made to serve the same end as the far more difficult mode of multiplying together the latter. Yet this peculiar property in logarithms is certainly no evidence of design, belonging, as it does, to the class of necessary truths, as much as that twice two are four. The pantheist argues, that it is the same in regard to those singular adaptations to circumstances we meet with so constantly in natural history.

In fact, notwithstanding the story told of Galen, I very much doubt whether the arguments of natural theology have ever yet converted an unbeliever. But, supposing this fundamental difficulty satisfactorily met, still the arguments of natural theology go only to prove certain of the Divine attributes, beneficence, wisdom, power—tending in no way whatever to show forth the equally glorious ones of holiness, justice, and mercy. In fact, the appropriate office of natural science in relation to religion is, not to convert the unbeliever, but to confirm the faith of one who already holds the truths of revelation, by opening to his view fresh instances of power, wisdom, and goodness, glorious in themselves, but still more glorious when, with the eye of the understanding thus supernaturally enlightened, we can trace in the phenomena and operations of nature a most close and wonderful analogy with the mysterious workings of Providence in the spiritual and unseen world, so that we may, without doing any violence to the words, apply to this an expression used in the Sacred Writings, in reference to a somewhat corresponding relation—"That which is glorious hath no glory in this respect, by reason of the glory which excelleth."

